# Continuous solutions of cosmic-rays and waves in astrophysical environments

**DOI:** 10.1038/s41598-023-48223-0

**Published:** 2023-12-21

**Authors:** K. Irshad, B. Ramzan, S. N. A. Qazi, F. Areeb, A. Rasheed, M. Jamil

**Affiliations:** 1 Department of Physics, University of Management and Technology, Lahore, 54770 Pakistan; 2 Department of Physics, Govt. College University, Faislabad, 38000 Pakistan; 3 Department of Physics, COMSATS University Islamabad, Lahore Campus, Lahore, 54000 Pakistan

**Keywords:** Astronomy and planetary science, Mathematics and computing, Physics

## Abstract

The propagation of energetic charged particles and cosmic rays in magnetized thermal plasma is focused. We consider a four-fluid system that consists of thermal plasma, cosmic rays, and two opposite propagating Alfvén waves to investigate the dynamics and energy exchange mechanisms of the system. Additionally, cosmic rays diffusion within the plasma is considered along the magnetic field lines whereas neglected the cross field line diffusion effects. This study is important for understanding of pressure gradients and their impact on the feedback in astrophysical environment. Over the last few decades, this problem becomes important when we discuss the interaction of cosmic rays with plasma in space, such as interstellar clouds or interstellar medium.

## Introduction

In the astrophysical environments the energy density of cosmic rays, components of the interstellar medium, different phases of gas (i.e., plasma) and magnetic field are of the same order of magnitude^1,2^. These components of the interstellar medium may interact with each other at the basis of equipartition of energy. Whereas the structural evolution of the interstellar and intergalactic mediums are mainly attributed to the dynamical role of the cosmic rays^3–5^. For instance, the Alfvén waves are triggered when the cosmic rays interact with thermal plasma. Cosmic rays may also be a result of continuous scattering of particles across the shock waves^6^. Shock wave structures are formed when maximum energy flux of the system is eaten up by the cosmic rays at the upstream state^7^ which can best be studied from hydro-magnetic approach^6,8,9^. If the plane shock waves progress through a plasma without any upstream precursor, they eventually reach to equilibrium state. The interaction of cosmic rays, thermal plasma, and Alfvén waves are treated from hydro-magnetic approach^10^, where a few closure parameters are restricted. Primarily hydrodynamics is considered a simple model that is, the two fluid model for shock waves caused by cosmic rays.

Two fluid model explores the effect of cosmic rays and background thermal plasma^11^. When Alfvén waves are added to two-fluid model, it would become a three or four-fluid model with two waves interfering strongly at the later end of the process. Under the multi-fluid theory, plasma, cosmic rays, and Alfvén waves(forward and backward) are all considered as distinct fluids^12^. The importance of cosmic rays and their coupling with the thermal plasma flow was highlighted by Ko^13^. He noted that the diffusion of cosmic rays through magnetic field depends on the strength of the coupling. Such dynamics are theoretically explored by either four-fluid or two fluid models. The two fluid model is applicable when the downstream states of the system are uniform whereas for the case of varying downstream states four-fluid model is used which is generally self-consisting^5^. The stochastic acceleration prevails the non-uniformity of the states preventing from converting four-fluid model into two-fluid model. For the non-linear test particle picture the four-fluid model provides the solution with far upstream ($P_c$ = 0) and far downstream ($P_w^+$ = 0) cases. Hamilton’s principle allows to understand mutual effect of a wave and its background on each other by incorporating the average hydro-magnetic Lagrangian density^14^. Propagating adiabatical pressure forms the system’s background and thus conserves the linear momentum, angular momentum, and energy. However, in the case of the superposition of many waves “weak turbulence” the adiabatic conservation equation is employed instead of the wave kinetic equation. Heavens et al. developed a hydrodynamic two-fluid model to formulate a self-consistent nonlinear theory of particle accelerated by the shock waves^15^. Schlickeiser et al. discussed in detail the quasi-linear transport equation for energetic charged particles in uniform magnetic field^16^. Zank et al. applied numerical simulation to study the cosmic-ray-mediated shock waves using the two-fluid model^17^. The spherical adiabatic and plane parallel shock waves were studied for the first time by them. They considered two types of shock waves one that that sweep up ambient magnetic field and other that interact with cosmic rays directly. One of the most striking aspects of astrophysical plasma system is that it often contains populations of particles that are not in thermodynamic equilibrium^18^. Ko et al. examined the impact of injection of shock waves structure^19,20^ modified by cosmic rays through multi-fluid model. Malkov et al. investigated the interaction between cosmic rays and two oppositely traveling Alfvén waves along with plasma as heat bath, and they found when the energy density of cosmic rays is comparable to the plasma, the cosmic rays have some effect on background plasma. However, it is very difficult to investigate coherently cosmic-ray system with a distribution function approach^21,22^. A cosmic ray plasma system allows only three types of energy exchange mechanisms, namely (i) work done by plasma through pressure gradients, (ii) cosmic ray streaming instabilities, (iii) stochastic acceleration. Through the interplay of these mechanisms, morphological structures are created in distinct ways.

Nonlinear test particle picture in shock wave background is examined by Ref.^23^. According to his idea, efficiency can be negative in a few regions, which means that the pressure of cosmic rays may be less downstream than the cosmic ray pressure without any shock. One changes from upstream to downstream smoothly but quickly while on the other hand, changes from downstream to upstream occur through discontinuity or sub-shocks. Moreover, gain in pressure of cosmic rays in the shock type flow occurs less frequently as compared to continuous flow. Here we are going to consider continuous solutions which are physically admissible. Moreover, we only consider super Alfvénic waves and these solutions contain both far upstream and far downstream regions. Certain parameters like magnetic flux, entropy constant, mass flux, total energy flux, momentum flux, and wave action integral are kept constant. As the plasma flow acts as a heat bath, we find that cosmic ray energy flux does not increase and therefore the efficiency comes from the density of cosmic rays or the density of pressure from the components namely cosmic rays, thermal plasma, forward and backward propagating Alfvén waves contained in the conservation of momentum equation. Depending on the compression ratio and the location of the sub-shock, the efficiency can be negative because the shock decelerates cosmic rays by some given factor. We adopt a four-fluid model, which consists of cosmic rays and two oppositely propagating Alfvén waves. We are considering Alfvén waves parallel to as well as opposite to magnetic fields, depending on the gradient of cosmic rays, we call them forward and backward propagating Alfvén waves^23^.

Alfvén waves propagation in the plasma is little tricky and there are usually two approaches involved namely (i) WKB and (ii) Non-WKB approximation. The choice of using either one of these approaches mainly depends on the conditions of characteristics length of plasma and wavelength of the Alfvén wave. Specifically, the major factor that determines in using one of these method to describe the Alfvén wave in any astrophysical environment, is the condition related to ratio of the Alfvén wavelength to characteristic scale length of plasma^24^ ($\lambda /L$). The Non-WKB method is best suited in the scenario in which the wavelength exceeds the characteristics scale length of plasma ($\lambda /L > 1$). Under this condition, the amplitude and other plasma parameters strongly fluctuate as compared to wavelength thus generating turbulence and non-uniformity in plasma. This also opens considerations for the involvement of wave reflection and couplings between waves in the given background^24,25^. Therefore, Non-WKB takes accounts for all these factors and provide better accurate description of the Alfvén wave propagation in the non-uniform plasma medium. For instance, studying the solar wind required Non-WKB Alfvén waves model considered as necessity approach because it is observed that wavelength size is comparable to solar radius whereas the scale length of the plasma is much smaller than the solar radius^24^. However, in our case we assume WKB approximation in treatise the Alfvén waves propagation in plasma background for several reasons. First, wavelength is much smaller than the scale length of plasma ($\lambda /L < 1$). Second, the turbulence condition is not taken into account in the model and therefore the amplitudes and other plasma parameter varies slowly as compared to wavelength. Finally, we do not consider the interaction or mixing between forward and backward waves and so under these circumstances WKB model is very appropriate in providing description of the dynamics of Alfvén waves.

We arranged the paper as follows. The four-fluid model of the cosmic-ray-plasma system is presented in “Numerical results” section. We studied the hydrodynamic model numerically using MATLAB R2020b and highlighted some important profiles in “Summary and discussion” section including increasing wave, shock wave type, and decreasing wave profiles. Finally in “Conclusion” section, we discuss and summarize these morphological structures, mentioning their implications related to astrophysical environments and also providing some concluding remarks.

## Four-fluid cosmic-ray plasma model

Skilling developed the equation for the cosmic-ray propagation^3,4^ and Dewar gave the energy exchange equation for them^14^. Whereas Ko combine both approaches and developed a four-fluid model for the cosmic-ray plasma system^13^, which includes two opposite propagating Alfvén waves, thermal plasma, and cosmic rays. The equations governing the model comprise (i) total mass flux, (ii) the energy equations for various different components (that is kinetic energy and thermal energy in the plasma, cosmic-ray energy, and wave energy), and (iii) magnetic flux. In uni-dimensional geometry and the components are treated as fluids described in terms of energy, pressure, and density. In this fluid system, mass density is carried by thermal plasma whereas waves and cosmic rays are treated as mass-less fluids with significant pressures. In preset study, uni-dimensional geometry is considered and assumed that there is no dissipation and the magnetic field is taken parallel to the plasma flow, the relevant governing equations are,

1 $$\begin{aligned}{} & {} \frac{\partial \rho }{\partial t}+\frac{\partial }{\partial x} (\rho U)=0, \end{aligned}$$

2 $$\begin{aligned}{} & {} \rho \frac{\partial U}{\partial t}+\rho U \frac{\partial U}{\partial x}=-\frac{\partial }{\partial x} (P_{th}+P_c+P^{+}_{w}+P_{w}^{-}), \end{aligned}$$

3 $$\begin{aligned}{} & {} \frac{\partial E_k}{\partial t }+\frac{\partial F_k}{\partial x}=-U\frac{\partial }{\partial x} (P_{th}+P_c+P^{+}_{w}+P_{w}^{-}), \end{aligned}$$

4 $$\begin{aligned}{} & {} \frac{\partial E_{th}}{\partial t}+\frac{\partial F_{th}}{\partial x}=U \frac{\partial P_{th}}{\partial x}, \end{aligned}$$

5 $$\begin{aligned}{} & {} \frac{\partial E_{c}}{\partial t}+\frac{\partial F_{c}}{\partial x}=[U+(e_{+}-e_{-})V_A] \frac{\partial P_{c}}{\partial x}+\frac{P_{c}}{\tau }, \end{aligned}$$

6 $$\begin{aligned}{} & {} \frac{\partial E_{w}^\pm }{\partial t}+\frac{\partial F_{w}^\pm }{\partial x}=U \frac{\partial P_{w}^\pm }{\partial x} \mp e_{\pm } V_A\frac{\partial P_{c}}{\partial x}-\frac{P_{c}}{2\tau }. \end{aligned}$$

Here $\rho$ and *U* represent the density and velocity of plasma respectively. In terms of kinetic energy, $P_k$, $E_k$ and $F_k$ represent as kinetic pressure, kinetic energy density and kinetic energy flux of plasma. For the cosmic-ray components, $P_c$, $E_c$, and $F_c$ are denoted as pressure, energy density, and energy flux respectively. Finally, for the wave component, it is described in terms of $P_{w}^{\pm }$, $E_{w}^{\pm }$, and $F_{w}^{\pm }$ denoted as pressure, energy density, and energy flux of waves respectively. (Here ± shows propagation of Alfvén waves in forward and backward direction). Alfvén speed is represented by $V_A=B$/$\sqrt{{\mu _0}{\rho }}$. When the field is in the same direction as the spatial coordinate, it’s uniform in uni-dimensional problems. In Eqs. (5) and (6), stochastic acceleration and cosmic-ray instability are represented by the terms $P_c/\tau$ and $e_\pm V_A\partial P_c/\partial x$. The energy fluxes for the four-fluid model are given by,

7 $$\begin{aligned}{} & {} F_k=E_kU, \end{aligned}$$

8 $$\begin{aligned}{} & {} F_{th}=(E_{th}+P_{th})U, \end{aligned}$$

9 $$\begin{aligned}{} & {} F_c=(E_c+P_c)[U+(e_{+}^{'} - e_{-}^{'}) V_A]-\kappa \frac{\partial E_c}{\partial x}, \end{aligned}$$

10 $$\begin{aligned}{} & {} F_{w}^\pm =E_{w}^\pm (U \pm V_A)+P_w^ \pm U. \end{aligned}$$

The energy density and pressure relation between components are given by,

11 $$\begin{aligned}{} & {} E_k=\frac{1}{2}P_k=\frac{1}{2}\rho U^2, \end{aligned}$$

12 $$\begin{aligned}{} & {} E_{th}=\frac{P_{th}}{\gamma _{g}-1}, \end{aligned}$$

13 $$\begin{aligned}{} & {} E_{c}=\frac{P_c}{\gamma _{c}-1}, \end{aligned}$$

14 $$\begin{aligned}{} & {} E_w^\pm =2P^\pm _w, \end{aligned}$$

where $\gamma _{g}$ and $\gamma _{c}$ denotes adiabatic index for thermal and cosmic-ray components. The simple model^4,20,26,27^ for the interaction between plasma, cosmic rays and waves are governed by three equations namely,

15 $$\begin{aligned}{} & {} e_\pm =e_\pm ^{'}=\frac{P_w^\pm }{P_w^+ + P_w^-}, \end{aligned}$$

16 $$\begin{aligned}{} & {} \frac{1}{\tau }=16\alpha \frac{V_A^2}{c^2} \frac{P_w^+ P_w^-}{P_w^{+}+P_w^{-}}, \end{aligned}$$

17 $$\begin{aligned}{} & {} \kappa =\frac{c^2}{3\alpha (P_w^{+}+P_w^-)}, \end{aligned}$$

where $e_\pm$ is the scattering frequency for forward and backward waves and $\kappa$ represents the diffusion constant. Finally the mass flux, magnetic flux, entropy constant, total flux energy, total pressure and wave action integral are expressed as

18 $$\begin{aligned}{} & {} \phi _B=B, \end{aligned}$$

19 $$\begin{aligned}{} & {} \phi _m=\rho U, \end{aligned}$$

20 $$\begin{aligned}{} & {} A=P_{th}\rho ^{-\gamma g}, \end{aligned}$$

21 $$\begin{aligned}{} & {} F_{tot}=F_{k}+F_{th}+F_c+F_w^{+}+F_w^{-}, \end{aligned}$$

22 $$\begin{aligned}{} & {} P_{tot}=P_{k}+P_{th}+P_c+P_w^{+}+P_w^{-}, \end{aligned}$$

23 $$\begin{aligned}{} & {} W_A=[F_c+\frac{(U+V_A)^2}{V_A}E_w^{+}-\frac{(U-V_A)^2}{V_A}E_w^-]. \end{aligned}$$

## Numerical results

For solving the four-fluid steady-state model (i.e., time dependency is neglected), we initially employ the parameters given by (7)–(23) into the general Eqs. (1)–(6) that govern the cosmic-ray plasma system. First order differential equations are derived in terms of pressure components (cosmic-ray, thermal plasma, forward and backward propagating Alfvén waves) and the velocity of the four-fluid system. Additionally, involving the cosmic-ray flux parameter (9) gives arise to two sets of the differential equations that include the diffusive flux term allowing it to dictate the mechanism for the cosmic-ray to get diffused within the plasma system. Standard set of ordinary differential equations is preferred and we have the following convenient set of equations. The set has seven variables (*x*, $U^2$, $P_{th}$, $P_c$, $P_w^{+}$, $P_w^{-}$, $\kappa \frac{dE_{c}}{dx}$), and seven ordinary differential equations to solve. Independent variable $``\xi ''$ is introduced to avoid the singularity and we chose ($M = 1$) when implementing the numerical scheme. Overall, this results in the set of seven autonomous ordinary differential equations as follows,

24 $$\begin{aligned} \frac{dx}{d\xi }= \frac{1}{2} \left( 1-M^{-2}\right) , \end{aligned}$$

25 $$\begin{aligned} \frac{dU^2}{d\xi } &=-\frac{1}{\rho }\frac{d P_c}{dx} \left[ \frac{e_+(1+\frac{1}{2}M_A^{-1})}{(1+M_A^{-1})}+\frac{e_-(1-\frac{1}{2}M_A^{-1})}{(1-M_A^{-1})}\right] \nonumber \\ & \quad +\frac{1}{\rho U}\left[ \frac{P_c}{2(1-M_A^{-2})\tau } \right] , \end{aligned}$$

where $M_A= \sqrt{\mu _0} \Phi _m/(\Phi _B\sqrt{\rho })=1/({{\tilde{\Phi }}\sqrt{\rho }})$ is the Alfvén Mach number.

26 $$\begin{aligned} \frac{dP_{th}}{d\xi }= -\frac{\gamma _{g}P_{th}}{2U^2} \left( \frac{dU^2}{d\xi }\right) , \end{aligned}$$

27 $$\begin{aligned} \frac{d P^\pm _w}{d\xi } &= -\frac{3 P^\pm _w}{2U^2}\frac{(1\pm \frac{1}{3}M_A^{-1})}{(1\pm M_A^{-1})}\frac{dU^2}{d\xi } \mp \frac{e_\pm M_A^{-1}}{2(1\pm M_A^{-1})}\frac{d P_c}{d\xi }\nonumber \\ & \quad -\frac{P_c}{4U(1\pm M_A^{-1})\tau }, \end{aligned}$$

28 $$\begin{aligned} \frac{dP_c}{d\xi }=\left( \frac{dP_c}{dx}\right) \left( \frac{dx}{d\xi }\right) , \end{aligned}$$

29 $$\begin{aligned} \frac{d}{d\xi }\left( \kappa \frac{dE_c}{dx}\right) &= \frac{U[1+(e_+-e_-)V_A]}{(\gamma _c-1)} \left( \frac{dP_c}{d\xi }\right) -\frac{P_c}{\tau } \left( \frac{dx}{d\xi }\right) \nonumber \\ & \quad +\frac{\gamma _c P_c}{(\gamma _c-1)} \frac{1}{2U}[1+\frac{1}{2}(e_+-e_-)M_A^{-1}] \left( \frac{dU^2}{d\xi }\right) \nonumber \\ & \quad +\frac{\gamma _c P_c}{(\gamma _c-1)}\frac{2V_A}{(P^+_w+P^-_w)}\left[ P^-_w\frac{dP^+_w}{d\xi }-P^+_w\frac{dP^-_w}{d\xi }\right] , \end{aligned}$$

30 $$\begin{aligned} \frac{dP_c}{dx}=\frac{(\gamma _c-1)}{\kappa }\left( \kappa \frac{dE_c}{dx}\right) . \end{aligned}$$

The Runge–Kutta approach in MATLAB is used to analyze and the equations are solved numerically. Just similar to^28^, here we are interested to find solutions for the four-fluid model that are (i) steady, (ii) continuous, (iii) physically admissible(for its criteria see^28^) and (iv) consists super-alfvénic flows(i.e $M_{A}=U/V_A > 1$). One of the criteria that are worth mentioning is that solution must approach towards the steady state for both upstream ($x\rightarrow -\infty$) and downstream ($x\rightarrow \infty$) cases. Here we vary different pressure components while keeping magnetic and mass flux constant, to examine the behavior of the solutions in terms of pressure components $P_{c}$, $P_{th}$, $P_{w}^{\pm }$ and velocity of plasma system and Alfvén waves as *U* and $V_A$ respectively. It results in some interesting profiles that give the unique morphological structure of the cosmic-ray plasma fluid system. Generally, these profiles are distinguished into three types^23^ (based on examining the behavior in the downstream region $x>0$) that are described as follows:

### Uniform and continuous profiles

**Figure 1:**
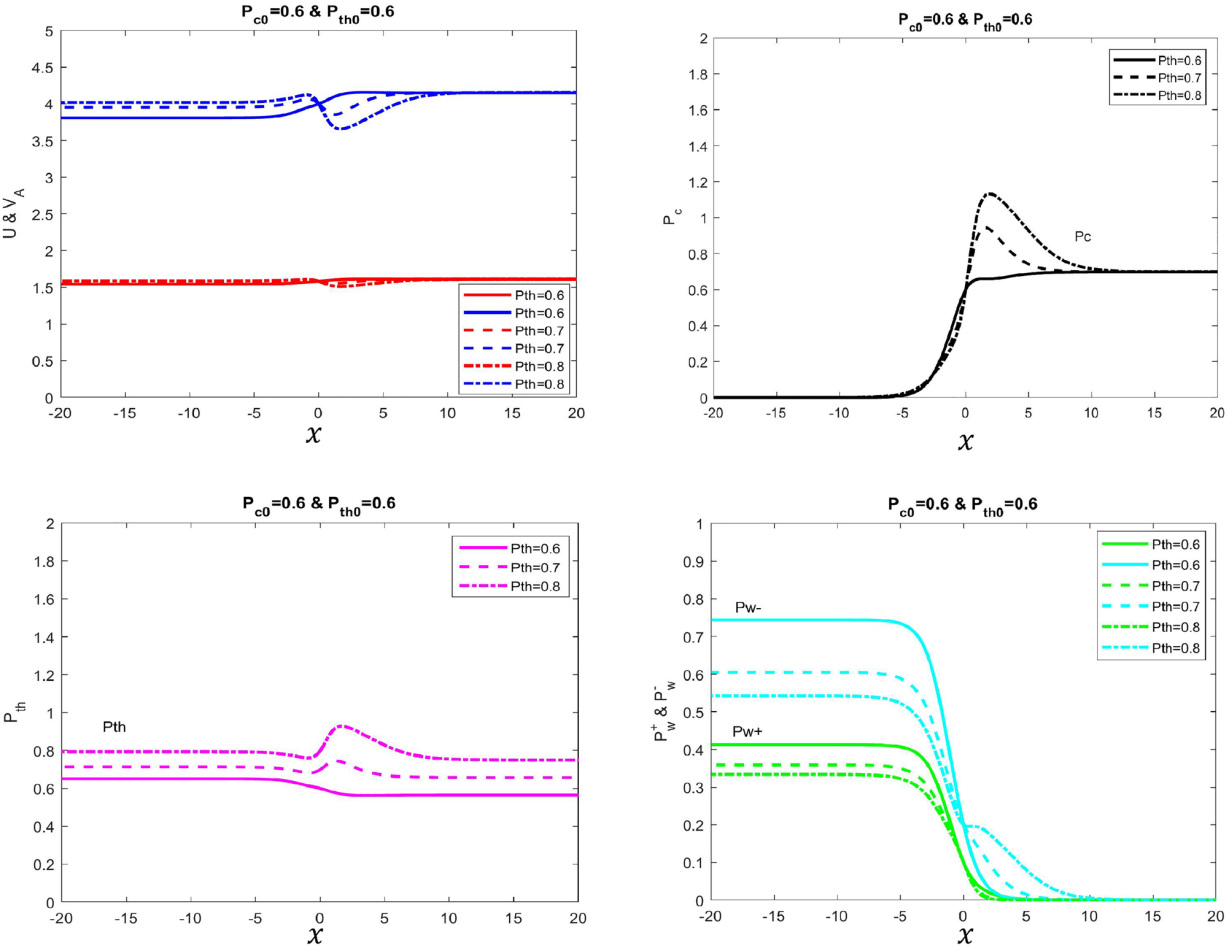
Velocity and different pressure profiles of four-fluid system with variations in thermal pressure is presented. In this example, $\gamma _\text{g}=5/3$, $\gamma _\text{c}=4/3$, ${\Phi _\text{m}}=1.6$, ${\Phi _\text{B}}=1$, $P_{c}=0.6$, $P^+_{w}=0.1$ and $P^-_{w}=0.2$. *Top Left Panel*: Shows velocity (Solid blue line with $P_{th}=0.6$, dashed blue line with $P_{th}=0.7$ and dotted-dash blue line with $P_{th}=0.8$) and Alfvén velocity (Solid red line with $P_{th}=0.6$, dashed red line with $P_{th}=0.7$ and dotted-dashed red line with $P_{th}=0.8$). *Top Right Panel* Shows cosmic ray pressure (Solid black line with $P_{th}=0.6$, dashed black line with $P_{th}=0.7$ and dotted-dash black line with $P_{th}=0.8$). *Bottom Left Panel* Shows thermal pressure (Solid Magenta line with $P_{th}=0.6$, dashed Magenta line with $P_{th}=0.7$ and dotted-dash Magenta line with $P_{th}=0.8$). *Bottom Right Panel* Shows forward (green color) and backward propagating Alfvén wave (cyan) pressures with different variations in thermal pressure. We tune $F_\text{tot}$ to obtain different solution curves.

For this given profile, we begin by setting the initial conditions of thermal and cosmic-ray pressure to be equal ($P_{c}=P_{th}=0.6$). While keeping other pressure components constant as shown in Table 1, we increase the $P_{th}$ by the interval of 0.1 (i.e Solid line $P_{th}=0.6$, dashed-dotted line $P_{th}=0.7$, dotted line $P_{th}=0.8$) to examine the behavior of the profile in terms of velocity and pressure components. In the upstream region, the *U* of the plasma flow increases accordingly with $P_{th}$ but eventually all the models converge asymptotically to a constant value at the later downstream region (Fig. 1 top-left). A similar behavior pattern is also observed for the $V_A$ of the waves as well. The cosmic-ray pressure $P_c$ elevates higher at the near origin ($x=0$) due to the increase of the thermal pressure $P_{th}$ in the system but later end at the downstream region, all the models converge asymptotically with the same constant value implying that system has reached toward its uniform state (Fig. 1 top-right). The pressure for both forward and backward waves $P_{w}^{\pm }$ are raised in accordance with the $P_{th}$ in the system but eventually, they decrease and die out at the downstream region (Fig. 1 bottom-right also see Table 1 for detailed parameter’s).

**Table 1:** Uniform and continuous solution.

Figure	$\phi _B$	$\phi _m$	$F_{tot}$	$P_{c0}$	$P_{th0}$	$U_{0}$	$P_{w0}^+$	$P_{w0}^-$
1.Solid line	1.0	1.6	29.99	0.6	0.6	16	0.1	0.2
2.Dotted line	1.0	1.6	30.20	0.6	0.7	16	0.1	0.2
3.Dashed-dotted line	1.0	1.6	30.80	0.6	0.8	16	0.1	0.2

### Shock wave type profiles

**Figure 2:**
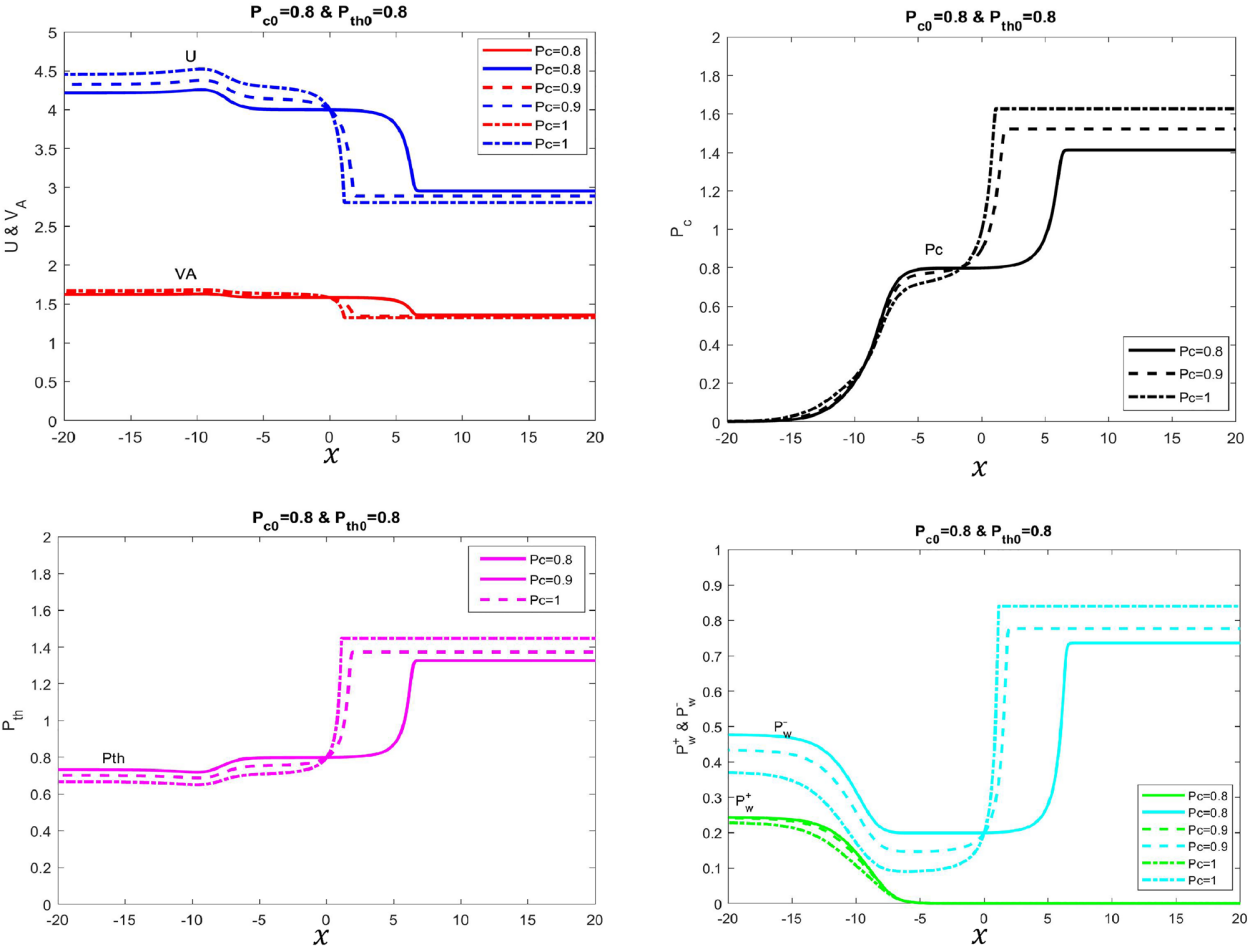
Velocity and different pressure profiles of four-fluid system with variations of cosmic ray pressure is presented. In this example, $\gamma _\text{g}=5/3$, $\gamma _\text{c}=4/3$, ${\Phi _\text{m}}=1.6$, ${\Phi _\text{B}}=1$, $P_{th}=0.8$, $P^+_{w}=0.1$ and $P^-_{w}=0.2$. *Top Left Panel* Shows velocity (Solid blue line with $P_{c}=0.8$, dashed blue line with $P_{c}=0.9$ and dotted-dash blue line with $P_{c}=1$) and Alfvén velocity (Solid red line with $P_{c}=0.8$, dashed red line with $P_{c}=0.9$ and dotted-dashed red line with $P_{c}=1$). *Top Right Panel* Shows cosmic ray pressure (Solid black line with $P_{c}=0.8$, dashed black line with $P_{c}=0.9$ and dotted-dash black line with $P_{c}=1$). *Bottom Left Panel* Shows thermal pressure (Solid Magenta line with $P_{c}=0.8$, dashed Magenta line with $P_{c}=0.9$ and dotted-dash Magenta line with $P_{c}=1$). *Bottom Right Panel* Shows forward (green color) and backward propagating Alfvén wave pressures (cyan) with different variations in thermal pressure. We tune $F_\text{tot}$ to obtain different solution curves.

Here we change the parameter of cosmic-ray pressure $P_{c}$ that overall generates shock-wave type structure in the cosmic-ray plasma system. Initially, we set the thermal and cosmic-ray pressure to be equal ($P_{c}=P_{th}=0.8$). But gradually, $P_{c}$ is varied by an interval of 0.1, while keeping other pressure components constant (shown in Table 2) to examine the velocity and pressure profiles of this system. As noticed from Fig. 2 (top-left), by raising $P_{c}$, both velocities *U* and $V_{A}$ become higher at the upstream region but lower at the downstream region (Fig. 2 top-left). Opposite trends are observed for pressure components$P_{th}$, $P_{c}$ and $P_{w}^{\pm }$ where they become lower at the upstream region and higher at the downstream region (Fig. 2 bottom-left and top-right). Apparently, the sudden surge or transition is observed between upstream and downstream at either side near the origin (i.e $0\le x \le 5$ shown in Fig. 2) thereby creating a larger width between these regions. Essentially, this important feature illustrates the creation of shock wave-type structures in the four-fluid system. Furthermore, raising $P_{c}$ increases the width thereby forming bigger shock wave-type structures. For the wave profiles as illustrated by Fig. 2 (bottom-right), both of their pressure is initially raised at the upstream region by the increase $P_{c}$ in the system. However, the pressure for both wave components falls and at later stages, the forward wave dies out completely leaving the remaining backward wave in the system. Finally, all the pressure and velocity components approach asymptotically to their constant values indicating that the cosmic-plasma fluid system has attained steady state condition (see also see Table 2 for detailed parameter’s).

**Table 2:** Shock wave type solutions.

Figure	$\phi _B$	$\phi _m$	$F_{tot}$	$P_{c0}$	$P_{th0}$	$U_{0}$	$P_{w0}^+$	$P_{w0}^-$
1.Solid line	1.0	1.6	30.30	0.8	0.8	16	0.000001	0.2
2.Dotted line	1.0	1.6	30.70	0.9	0.8	16	0.000001	0.2
3.Dashed-dotted line	1.0	1.6	30.90	1	0.8	16	0.000001	0.2

### Monotonically increasing and continuous type profiles

**Figure 3:**
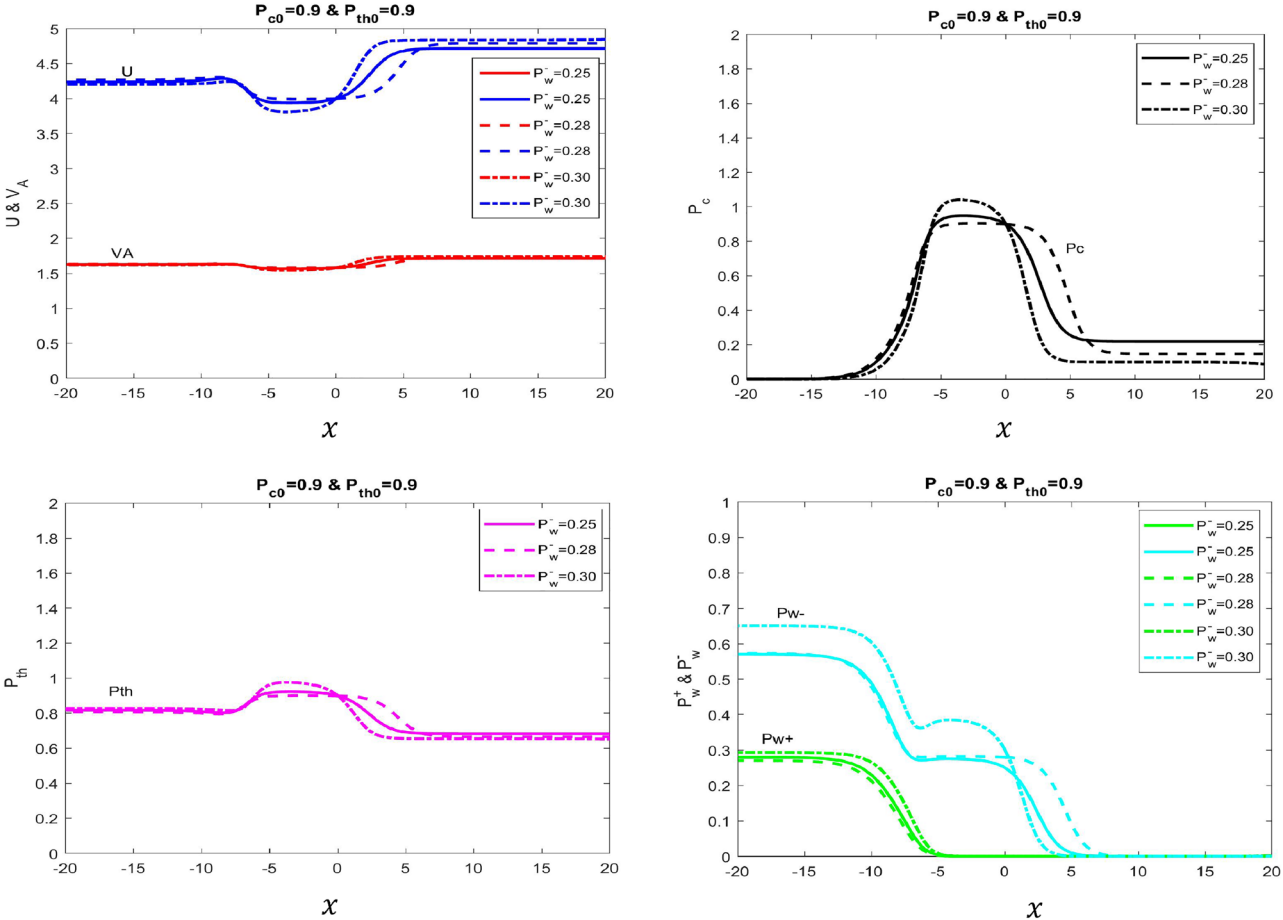
Velocity and different pressure profiles of four-fluid system with variations of backward propagating wave pressure is presented. In this example, $\gamma _\text{g}=5/3$, $\gamma _\text{c}=4/3$, ${\Phi _\text{m}}=1.6$, ${\Phi _\text{B}}=1$, $P_{th}=0.8$, $P^+_{w}=0.1$ and $P^-_{w}=0.2$. *Top Left Panel* Shows velocity (Solid blue line with $P^-_{w}=0.25$, dashed blue line with $P^-_{w}=0.28$ and dotted-dash blue line with $P^-_{w}=0.30$) and Alfvén velocity (Solid red line with $P^-_{w}=0.25$, dashed red line with $P^-_{w}=0.28$ and dotted-dashed red line with $P^-_{w}=0.30$). *Top Right Panel* Shows cosmic ray pressure (Solid black line with $P^-_{w}=0.25$, dashed black line with $P^-_{w}=0.28$ and dotted-dash black line with $P^-_{w}=0.30$). *Bottom Left Panel*: Shows thermal pressure (Solid Magenta line with $P^-_{w}=0.25$, dashed Magenta line with $P^-_{w}=0.28$ and dotted-dash Magenta line with $P^-_{w}=0.30$). *Bottom Right Panel* Shows forward (green color) and backward propagating Alfvén wave (cyan) pressures. We tune $F_{tot}$ to obtain different solution curves.

We change the backward-wave pressure parameter $P_{w}^{-}$ while keeping other pressure and velocity variables constant as depicted in Table 3. As illustrated in Fig. 3 (top-left), the general trend for the velocity profile in terms of *U* and $V_{A}$ shows that it is lower in the upstream region and higher in the downstream region. Increasing the $P_{w}^{-}$ allows to raise the velocity in the downstream region. In contrast, the pressure profile for the thermal system as shown in Fig. 3 (bottom-left) displays the opposite behavior as it is higher in the upstream region and lower in the downstream region. This implies that pressure is decreasing and increasing $P_{w}^{-}$ causes further lower levels of thermal pressure in the cosmic-ray fluid system as illustrated in Fig. 3. The noticeable feature in this profile is that prior to the downstream region ($-5\le x\le 0$), the system temporarily is in a uniform state in terms of velocity and pressure profiles. Since the backward wave $P_{w}^{-}$ is interlinked with other components in the fluid system, its behavior brings a direct impact to them as well. So its steady-state value keeps the system overall in a uniform state. However, as it shows decreasing behavior in the downstream region, the pressure for cosmic-ray and thermal plasma correspondingly decreases accordingly thus giving rise to the velocity of the fluid in the system. But eventually, the cosmic-ray fluid system in terms of velocity and pressure reaches to steady state in a far downstream region (Fig. 3 bottom-right also see Table 3 for detailed parameter’s).

**Table 3:** Monotonically increasing and continuous type solution.

Figure	$\phi _B$	$\phi _m$	$F_{tot}$	$P_{c0}$	$P_{th0}$	$U_{0}$	$P_{w}^+$	$P_{w}^-$
1.Solid line	1.0	1.6	32.90	0.9	0.9	16	0.000001	0.25
2.Dotted line	1.0	1.6	33.0	0.9	0.9	16	0.000001	0.28
3.Dashed-dotted line	1.0	1.6	33.60	0.9	0.9	16	0.000001	0.30

## Summary and discussion

In this article, we have studied cosmic ray propagation in the plasma and examined the flow structure by using the approach of the hydrodynamic model. Here, we consider the four fluid system that comprises cosmic rays, thermal plasma, and forward and backward propagating self-excited Alfvén waves that provide the description of cosmic-ray plasma structure. Here we have explored the energy exchange mechanism between components and examined the effects on the dynamics and morphological structure by altering the pressure components in the system, namely $$P_{c}, P_{th}, P_{w}^{-}$$ while keeping $$ P_{w}^{+}$$ constant in all three profiles. Essentially, three distinct profiles are produced that tend to reach steady-state at both far upstream and downstream, and each of the profile exhibits a distinct plasma structure based on the energy exchange mechanism that is discussed as follows:

### Uniform and continuous profiles

In these profiles, the thermal pressure is changed within the regime 0.6 $\le$ $P_{th}$ $\le$0.8. Generally, the profile displays non-monotonic behavior in terms of *U*, $V_{A}$, $P_{c}$, and $P_{th}$ in the downstream region as illustrated in Fig. 1. Initially, the stochastic acceleration governs the energy mechanism in the fluid at the region $(-5<x<3)$ (shown in Fig. 1 at the top and bottom-right) where the energy is transferred from the waves to cosmic rays. As a consequence, the pressure for both waves tends to decrease $(\frac{\partial P_{w}^{\pm }}{\partial x}<0)$ and at the same time it increases for cosmic rays $(\frac{\partial P_{c}}{\partial x}>0)$. This mechanism accounts for the behavior of cosmic rays to undergo excitation and acceleration that results in to increase its pressure within the fluid. Eventually, at the critical point ($x=3$) where the forward wave dies out completely while the backward wave remains, the system is now reduced towards a three-fluid model. At this stage, the cosmic-ray pressure reaches the optimal value, and from here onward, work done by plasma takes control of the energy mechanism in the system. As the pressure gradient of the thermal plasma becomes less $(\frac{\partial P_{th}}{\partial x}<0)$ illustrated in Fig. 1(bottom-left), both pressures for cosmic rays and backward waves tend to decrease as well. At the later downstream region, when the backward wave dies out completely, the $P_{c}$ tends to reach asymptotically to its steady state value. Another important observation shows that raising thermal pressure $P_{th}$ directly causes to increase in $F_{th}$ that enhances the energy exchange mechanism process for the cosmic rays. The speed flow of the fluid *U* is directly impacted by the pressure gradients in the system. As illustrated in Fig. 1(top-left), the velocity profile is updated along the upstream as well as downstream regions due to the overall contribution by the pressure gradients of thermal, cosmic rays, and self-excited Alfvén waves dictated in the momentum Eq. (3). For instance, the *U* decreases due to the effect of the increasing behavior of the pressure gradient for both thermal $(\frac{\partial P_{th}}{\partial x}>0)$ and cosmic-ray $(\frac{\partial P_{c}}{\partial x}>0)$ and vice-versa that generates overall non-monotonic behavior at near downstream region. Meanwhile, as the thermal pressure gradient rises, the plasma density in the system becomes denser and this causes to slow the speed down in the system $(\frac{\partial U}{\partial x}<0)$. On the other hand, the fall of thermal pressure results in the plasma density becoming diluted and increase the speed of the system $(\frac{\partial U}{\partial x}>0)$. A similar behavior pattern can also be described for the Alfvén speed as well. Eventually, at the far downstream region, the pressure and velocity profiles of the plasma system reach the uniform state where all of the given parameters reach their same constant asymptotic value respectively.

### Shock wave type profiles

In these profiles, the cosmic-ray pressure is changed within the regime 0.8 $\le$ $P_c$ $\le$ 1 that generates a sub-shock-like structure in the multi-fluid system. Overall, the profile displays monotonic behavior regarding velocity and pressure profiles. Normally at the beginning, the stochastic acceleration dominates the energy mechanism in the upstream region($- 15\le x \le -6$). In this usual routine, the stochastic acceleration term $\frac{P_{c}}{\tau }$ allows energy exchanges between waves and cosmic rays that cause the pressure gradient for the former and latter to go down and up respectively. In this retrospect, the cosmic rays get diffused within the plasma system dictated by the diffusive flux parameter “$\kappa$” term in the cosmic ray flux equation. However, after the disappearance of the forward wave component, the system transits to a temporary steady-state condition for both velocity and pressure profiles around at mid-region between the end of the upstream ($x>-6$) and starting of the downstream region(depending on the $P_c$) as illustrated in Fig. 2. Then suddenly the pressure gradient for both cosmic rays and backward wave rises again at the downstream region. This increasing behavior is due to the cosmic-streaming instability that governs the energy mechanism in this regime. In this scenario, cosmic rays excite the Alfvén waves, and hence the streaming instability term $e_\pm V_A\partial P_c/\partial x$ remains as dominant effect in the pressure Eqs. (5) and (6) that causes to increase $P_{c}$ and $P_{w}^{-}$ for both cosmic-rays and backward-wave component in the plasma system respectively. Consequently, the cosmic rays via waves transfer the energy and momentum to thermal gas that generates more thermal pressure $P_{th}$ in the system. Overall, the effect of streaming instability causes pressures for all components to increase monotonically in the downstream region. Meanwhile, the combination behavior for all the pressure components causes the speed flow of the plasma to decrease monotonically throughout the upstream towards the downstream region as shown in Fig. 2 (top-left). More importantly, the sudden transition decrease of the velocity between at downstream region ($0<x<3$) creates a width gap between the upstream and downstream regions. This strongly indicates the generation of shock-like structures due to the presence of accelerating cosmic rays in the regime. It is observed that raising $P_{c}$ enlarges the width gap and narrows the temporary steady-state region and this strongly indicates the growth of a shock-wave like structure due to the increasing number of cosmic rays getting accelerated and diffused within the system. Finally, the cosmic-ray plasma system is reduced to three fluid system when it reaches to uniform state, which now only consists of surviving back-ward waves at far downstream region.

### Monotonically increasing and continuous type profiles

In these profiles, the back-ward wave pressure is changed within the regime 0.8 $\le P_{w}^{-} \le$ 1 that overall produces increasing monotonic behavior of the velocity profile in the downstream region as illustrated in Fig. 3 (top-left). Once again, stochastic acceleration plays role in the upstream region that simultaneously causes the pressure gradients for cosmic rays and thermal gas (Alfvén waves) to rise (fall) respectively ($-15<x<-5$ illustrated in Fig. 3 top and bottom-left and right). The encompassing behavior for these pressure gradients directly causes the velocity *U* of the plasma fluid to go down in the upstream region. After the disappearance of the forward wave, the cosmic-plasma fluid reaches a temporary stationary state near the end of the upstream region $(-5<x<0)$ in which both velocity and pressure profile remain constant. At the downstream region, the energy mechanism is governed by work done by plasma against the pressure gradients that result in the decrease of pressure profile ($P_{c}$, $P_{th}$, $P_{w}^{-}$) in the system. In this scenario, cosmic rays are getting decelerated due to work done by plasma that eventually causes the decline of all other pressure components in the plasma fluid. This overall decreasing behavior updates the increasing trend of the velocity profile, clearly showing that both *U* and $V_{A}$ are increasing monotonically. Finally, when the system attains a uniform state at the far downstream region, all velocity and pressure variables asymptotically reach towards constant value respectively. Furthermore, the four-fluid model for the cosmic-ray plasma system is now simply reduced to a two-fluid model.

### Implications of the study

Here we present the implications of some important results in the astrophysical environments. Amongst three profiles, the shock wave type profile due to its characteristics carries great significance in describing several astrophysical related observations. As argued by Wang et al.^29^ the shock wave type model can be applicable to interpret the observations of the supernova remnants and acceleration the cosmic-rays while not considering their applications to specific present cases. Generally in shock wave type scenario, the cosmic-ray streaming instability involves the interaction of cosmic-rays with the thermal plasma thereby generating the excitation of the hydro-magnetic waves producing as Alfvén waves in the system. Since the gyro-resonant scattering causes strong fluctuations in the magnetic fields, the high intensity perturbations in the hydro-magnetic waves causes to emit the synchrotron radiations in the form of gamma rays and x-rays. Such highly energetic radiations are possibly observed in the galactic disk in the form of Fermi Bubbles structures (see also^30–32^) and supernova remnants (see also^29,33^). According to Wang et al.^29^ at the supernova remnants shocks, the diffusive flux term has to be small enough in order to observe $H\alpha$ line that prevents the complete ionization of the hydrogen atom. Our model is in close agreement with the observation, that the diffusive flux term indeed tends to decrease at far downstream region and becomes smaller after the shock effects. As the diffusive flux term is inversely proportional to both coupling strength and Alfvén waves pressure, the rise of cosmic-ray pressure during shock at downstream region allows more number of cosmic-rays to be diffused within the thermal plasma system. This enhances the coupling between cosmic rays and thermal plasma that causes the diffusive flux term to decrease at the later downstream region. Similarly, the rise of backward wave pressure causes the diffusive flux to become smaller after the shock effects at downstream region. So our shock wave like profiles closely corresponds and agree with the observation of small diffusive flux term found at the supernova remnants.

## Conclusion

Cosmic rays keep great importance in astrophysical environments. In the interstellar or intergalactic medium, cosmic rays may attain comparable energy densities and pressure with magnetic field and the thermal plasma. As the cosmic rays are the highly charged particles when they start propagating into the ISM, it gets trapped by chaotic magnetic fields that allow interaction and coupling with other components available in the surrounding via gyro resonant interaction. In this manner, cosmic rays play a pivotal role in governing the structure and dynamics of the interstellar medium. In using the hydrodynamic approach, we have extensively examined the energy exchange mechanism between cosmic rays and thermal plasma. By inspecting several morphological solutions, we categorize them into two types namely test particle and shock-wave like solution. In a test particle picture scenario, usually, the four-fluid model is simply reduced towards a two-fluid system in which both forward and backward waves die out at far-down-stream regions and are eventually left with thermal plasma and cosmic rays in the system. However, in the case of a shock-wave like solution, the backward wave survives in the system imploring that the four-fluid model transits towards the three-fluid model. This shock-wave-like behavior carries great importance as this can be applicable in an interstellar environment to study the interaction between cosmic rays and thermal plasma. For instance, when the cosmic rays interact with diffuse molecular clouds to produce Alfvén waves, the shock-wave type solutions could possibly provide insights into how these self-excited waves can generate instability in the molecular clouds. In the presence of strong damping effects in the clouds, the mechanism itself can drive the waves to heat the cloud enormously. In this process, it can be explored how the heating by the waves can lead towards instability to further drive the star formation in molecular clouds. Another astrophysical scenario where the hydrodynamic model can be applied is the study of the galactic winds from the center of the galaxy. In this context, it can be studied how the morphology of the plasma wind changes as it moves against the external gravity in the galactic system.

### Supplementary Information

Supplementary Information.

## Data Availability

Matlab R2020a is used for the numerical analysis of the results present in this paper. Any Equation fits or plotting can be provided on a reasonable request to the corresponding author at bilal.ramzan@umt.edu.pk.
