# Supplementary material for: Continuous solutions of cosmic-rays and waves in astrophysical environments
Source: Sci Rep. 2023 Dec 21;13:22850. doi: 10.1038/s41598-023-48223-0 (PMC10739817; doi:10.1038/s41598-023-48223-0)
Supplement: Supplementary file 1 — Supplementary Information. [file 41598_2023_48223_MOESM1_ESM.pdf]

Energy Exchange Mechanisms

The evolution and morphological structure of cosmic-ray plasma system in the four fluid model depends on the interplay of the energy exchange mechanisms between cosmic-rays, thermal plasma, forward and backward propagating Alfv\\'en waves components. The energy exchange mechanisms between these components involves three distinct types of energy transfer process namely (i) work done by plasma against pressure gradients $UdP_c/dx, UdP_w^\pm/dx$ (ii) cosmic-ray streaming instability (iii) stochastic acceleration.

Most importantly, the cosmic ray-diffusive flux plays critical role to provide transport and energy facilitation of cosmic-rays in the thermal plasma. As depicted by $\kappa \frac{\partial E_c}{\partial x}$ in Equation (29) , the plot of diffusive flux term in Figure~\ref{Energy Exchange Mechanism} {\it Left Panel Green color}: carries the information of the convection and cosmic-ray streaming instability of the cosmic-rays while undergoing diffusion in the system. The Figure: Supplementary {\it Left Panel and Right panel}: illustrates the various energy exchanges that takes places between the components and its effects in the progression of the total pressure in the system for the shock wave-type profile. Initially at the far upstream region $(-20<x<-5)$, stochastic acceleration clearly dominates the energy transfer mechanism as it is evident by the decreasing and increasing trend for the work done by plasma on forward/backward propagating waves and cosmic-rays respectively. But the rise of diffusive flux primarily boosts the increasing behavior the work done by plasma on cosmic-rays and so it provides major contribution in accelerating the cosmic-rays in the plasma system. However, at the later upstream region $(-10<x<-5)$, the further decline of forward wave directly causes the diffusive flux to go down until both of them vanish and this indicates the halt of stochastic acceleration. As a consequence, the work done by plasma on backward wave and cosmic-rays becomes saturated that results plasma system to reach temporary stable state at between near upstream and downstream region $(-5<x<2)$. Following at the later upstream region $(2<x<5)$, the sudden rise of both cosmic-ray diffusive flux and work done by plasma on backward-wave triggers the surge of the work done by plasma on cosmic-rays and thereby creating sub-shock like structures. But then finally the growth of the energy feedback to cosmic-rays and backward wave ceases $(x=7)$ when the diffusive-flux diminishes and vanishes completely thereby resulting the cosmic-ray plasma system to reach uniform state at far downstream region.

The energy exchanges mechanisms between components directly influences and updates the total pressure/momentum flux in the plasma system as indicated in the Figure Supplementary {\it Left Panel}. Considering the total pressure by thermal component alone, it is observed that at near downstream region $(2<x<5)$, the effects of shock type gives slight rise to thermal

pressure component. Adding the pressure gradients by the other components modifies the evolution of the momentum flux for the four-fluid system in two ways. Firstly, it raises the level of the total pressure due to the involvement of the of the behavior effect by the pressure components due to cosmic-rays, forward and backward propagating Alfv\'en waves. It is noted that pressure gradient by forward wave slightly contribute the update of the total pressure as it is declining and dying out at earlier stages at upstream region. But the pressure contributions by the cosmic-rays and backward wave significantly raises the levels of the momentum flux in the four-fluid system. Second, the cosmic-ray and backward wave pressure components provides sudden increase to the total pressure of the system at the given two distinct upstream $(-10<x<-5)$ and downstream regions $(2<x<5)$ respectively. This obvious trend is explained by the presence of diffusive-flux, as it is directly responsible to increase the pressure of cosmic-rays and backward waves especially at downstream region. So, by adding the pressure components of cosmic-rays and backward waves, one can see how it promotes the growth of total momentum flux. As a consequence, it enlarges the band width leading to creation of shock like structure at this given particular regime. Finally, at far downstream region the total momentum flux with the presence of the three pressure gradients remain constant to show that plasma fluid system has reached towards steady state. In the nutshell, the complex behavior of energy-exchange mechanism and total momentum-flux clearly depicts the evolution of the four-fluid that gets reduced towards three-fluid system and results shock-like picture for this given scenario.

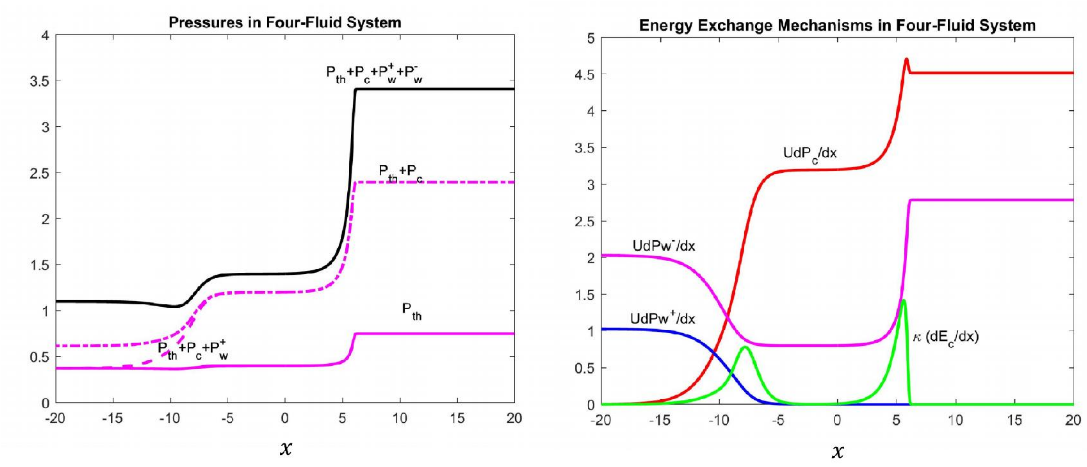

Figure: Energy Exchange Mechanisms
